# Theoretical Study of *p*-Block Metal Single-Atom-Loaded Carbon Nitride Catalyst for Photocatalytic Water Splitting

**DOI:** 10.3390/molecules29092030

**Published:** 2024-04-28

**Authors:** Mengning Chen, Yidi Wu, Qiang Wan, Sen Lin

**Affiliations:** 1 State Key Laboratory of Photocatalysis on Energy and Environment, Fuzhou University, Fuzhou 350116, China; cmn211320315@163.com (M.C.); wuyiddd519@snnu.edu.cn (Y.W.); 2 Key Laboratory of the Ministry of Education for Advanced Catalysis Materials, Institute of Physical Chemistry, Zhejiang Normal University, Jinhua 321004, China

**Keywords:** graphitic carbon nitride, *p*-block metal, water splitting, semiconductor photocatalysis, DFT

## Abstract

Graphitic carbon nitride (*g*-C_3_N_4_), recognized for its considerable potential as a heterogeneous photocatalyst in water splitting, has attracted extensive research interest. By using density functional theory (DFT) calculations, the regulatory role of *p*-block metal (PM) single atoms on the photocatalytic activity of *g*-C_3_N_4_ in overall water splitting was systematically explored. The incorporation of PM atoms (Ge, Sn and Pb) led to a reduction in the overpotentials required for both the oxygen evolution reaction (OER) and the hydrogen evolution reaction (HER). Combined with the electronic structures analysis via hybrid functional, it was found that the introduction of Ge, Sn or Pb optimizes the positions of the valence band maximum (VBM) and the conduction band minimum (CBM), providing a robust driving force for HER and ensuring substantial driving force for OER. Meanwhile, the presence of these three PMs induces the spatial separation of VBM and CBM, inhibiting the recombination of carriers. These findings have significant implications for the design and preparation of efficient photocatalysts.

## 1. Introduction

Over the past five decades, environmental pollution and the energy crisis have emerged as significant global challenges. The pursuit of harnessing solar energy and achieving photocatalytic water splitting into oxygen and hydrogen have been regarded as a scientific holy grail. This process offers an environmentally friendly alternative to conventional energy sources by producing clean energy without emitting pollutants, thereby garnering substantial global interest [1,2,3,4,5,6]. Despite considerable advancements in this field [7,8,9,10], the development of effective photocatalysts for overall water splitting retains formidable challenges [11,12], such as low utilization efficiency of visible light, low stability and short catalyst life under high oxidative conditions. Photocatalysts can be divided into homogeneous [13,14,15] and heterogeneous [16], the former usually being organic molecules with suitable HOMO-LUMO positions and energy gaps, while the latter are usually inorganic semiconductors with suitable bandgap and band edge positions. Unfortunately, even though homogeneous photocatalysts have the advantage of high selectivity and high activity, they still have some non-negligible drawbacks, such as high cost, high toxicity, scarcity in nature and instability under reaction conditions. Essentially, an ideal heterogeneous photocatalyst for water splitting should exhibit several key attributes: solution stability, a suitable bandgap (*E*_g_) of 2–3 eV that balances driving force and visible light utilization and high economic efficiency. Of particular importance is that the CBM and VBM of the photocatalyst must match the redox potentials of the reactions involved in the water splitting process, i.e., covering the entire range of redox potential between OER and HER [17,18,19,20,21].

Graphitic carbon nitride (*g*-C_3_N_4_), a notable metal-free photocatalyst similar to traditional inorganic photocatalysts (e.g., TiO_2_, MoS_2_, BiVO_4_) [22,23], has garnered significant attention due to its exceptional capability in hydrogen production using water and visible light [24]. However, its broader application is hindered by challenges such as a low response to visible light, a high recombination rate of photoinduced electron-hole pairs and the lack of surface active centers [25]. As a result, numerous studies have been conducted to enhance the photocatalytic properties of *g*-C_3_N_4_ [26,27,28]. These include surface modification [29,30], construction of heterostructures [31] and introduction of heteroatoms [32,33]. For instance, Tang et al. prepared non-metallic atoms co-doped with *g*-C_3_N_4_ and optimized the photocatalytic performance of *g*-C_3_N_4_ [34]. These studies suggest that the doping strategy can directly influence the performance of photocatalysts via tuning the VBM and/or CBM.

In the doping strategy, the modification of traditional photocatalysts through the incorporation of atomically dispersed noble metal is recognized for its superior atomic efficiency and broad application potential. This approach, termed single-atom photocatalysing, has been employed for water splitting [35,36,37,38,39,40]. For instance, Liu et al. encapsulated Pt_1_ within *g*-C_3_N_4_, a modification that significantly enhanced HER performance and resulted in a reaction rate ten times greater than that of Pt nano-particles in *g*-C_3_N_4_ [41]. To minimize the reliance on precious metal elements, *p*-block metals (PM) such as Ga, In and Sn, have emerged as alternative dopants in the modification strategy of *g*-C_3_N_4_-based water splitting photocatalysts due to their abundant reserves. Sun et al. demonstrated that doping *g*-C_3_N_4_ with Ga results in substantial improvement (~162-fold) in HER performance [42]. Furthermore, Yang et al. discovered that incorporating In into *g*-C_3_N_4_ expedited the separation and migration of photogenerated carriers compared to undoped *g*-C_3_N_4_ [43]. Rouby et al. further illustrated that doping Sn into *g*-C_3_N_4_ markedly augmented the absorption of solar light and facilitated the separation of photogenerated carriers, thereby enhancing photocatalytic OER activity [44].

Despite the successful enhancement of photocatalytic water splitting performance by non-precious metals in *g*-C_3_N_4_, a systematical investigation into the regulation effects of various non-noble metals on the overall water splitting performance of *g*-C_3_N_4_ is still lacking, which is pivotal for the rational design efficient photocatalysts of water splitting. By employing density functional theory (DFT) calculations, we investigated *g*-C_3_N_4_ doped with various PMs from the III A, IV A and V A group (PM = Al, Ga, In, Tl, Ge, Sn, Pd, Sb and Bi), denoted as PM-C_3_N_4_. Calculated energy diagrams for OER and HER revealed that the reactivity of PM-C_3_N_4_ (PM = Ge, Sn and Pb) was enhanced and was a potential candidate for photocatalytic overall water splitting. More importantly, with the introduction of PM (PM = Ge, Sn and Pb), though their *E*_g_ changes slightly, the VBM and CBM shift properly, leading to an enhanced driving force for OER while sacrificing little driving force for HER. These findings indicated significant implications for the design and fabrication of highly efficient photocatalysts.

## 2. Results and Discussion

### 2.1. Structures and Stability

**Figure 1:**
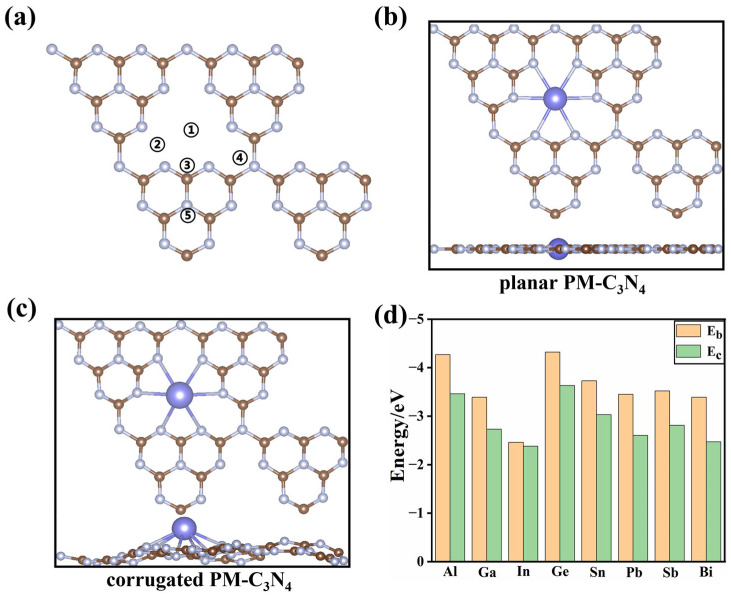
(**a**) Five possible sites for PM_1_ loading on the *g*-C_3_N_4_ surface. (**b**) Top view and side view of the structure of planar PM-C_3_N_4_ (PM = Al, Ga, In, Ge, Sn, Pb, Sb and Bi). (**c**) Top view and side view of the structure of corrugated PM-C_3_N_4_ (PM = Tl). Color scheme: greyish-white, nitrogen; brown, carbon; blue, PM_1_. (**d**) Binding energy ($E_{b}$) of various PM_1_ supported by *g*-C_3_N_4_ and corresponding cohesion energy ($E_{c}$) for the unitcell of PM bulk.

For single-atom catalysts, it is imperative to maintain effective interaction between individual atoms and the substrate, thereby ensuring atomic dispersion and preventing aggregation. Consequently, the stabilities of various PM-C_3_N_4_ (PM = Al, Ga, In, Tl, Ge, Sn, Pd, Sb and Bi) were first analyzed. For each PM, five potential adsorption sites were evaluated (Figure 1a). The configuration exhibiting the highest binding energy was selected for further investigation, with optimized configurations are shown in Figure S1. The findings revealed that all PMs were most stable at the 6-fold cavity of *g*-C_3_N_4_ (① in Figure 1a). In most cases, the introduction of PM_1_ would not change the planar structure of *g*-C_3_N_4_ (Figure 1b) except for the case of PM = Tl. It can be seen from Figure 1b that the *g*-C_3_N_4_ underwent significant deformation after doping with Tl, resulting in a corrugated Tl-C_3_N_4_ (as shown in Figure 1c). Due to the significant structural differences between Tl-C_3_N_4_ and other PM-C_3_N_4_, Tl-C_3_N_4_ is excluded to avoid the introduction of additional geometric factors that make it difficult to objectively analyze the key factors that regulate the catalytic performance in the doping strategy. For the planar PM-C_3_N_4_, PM_1_ forms six PM–N bonds, in the range of 2.35 to 2.45 Å (Table S1). The PM-N bond length increases as the period number of PM_1_ increases, which can be attributed to the increasing ionic radius of PM with increasing period number. In addition, it can be seen from Figure 1d that for elements of the same group, the negative values of *E_b_* and *E_c_* of the PM become smaller as the period number of the element increases and the value of *E_b_* is smaller than the corresponding *E_c_*. More importantly, *E*_b_ is more negative than *E_c_* for all PM_1_, indicating their thermal stability on *g*-C_3_N_4_; the high binding strength would prevent them from suffering agglomeration.

### 2.2. Photocatalytic Water Splitting Reaction

#### 2.2.1. OER Reactivity

Based on the above investigation in terms of stability, the photocatalytic reactivity of eight PM-C_3_N_4_ (PM = Al, Ga, In, Ge, Sn, Pb, Sb and Bi) towards overall water splitting reaction was further investigated. The overall water splitting reaction can be expressed in terms of the following equation:

2H_2_O → 2H_2_ + O_2_, (1)

which can be separated into two half-reactions, involving the oxygen evolution reaction (OER) and hydrogen evolution reaction (HER). Herein, the OER process was first investigated. The overall OER reaction equation can be written as follows:

2H_2_O → O_2_ + 4H^+^ + 4e^−^, (2)

**Figure 2:**
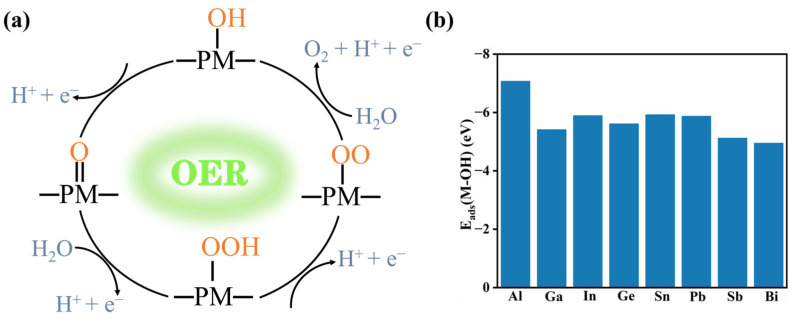
(**a**) Mechanism diagram of the proposed photocatalytic OER with PM-C_3_N_4_. (**b**) The adsorption energies of OH intermediate adsorbed on metal single atoms of PM-C_3_N_4_.

One can find that the overall OER involves the transfer of four electrons, and the overall process can take place via four one-electron elementary steps as follows [45,46,47] (Figure 2a):

*OH → *O + H^+^ + e^−^, (3)

*O + H_2_O → *OOH + H^+^ + e^−^, (4)

*OOH → *OO + H^+^ + e^−^, (5)

*OO + H_2_O → *OH + O_2_ + H^+^ + e^−^, (6)

where the * refers to the adsorption site on PM-C_3_N_4_. Interestingly, the H_2_O molecule tends to spontaneously dissociate into *OH and *H intermediates at the PM_1_ site, suggesting that the PM_1_ site of PM-C_3_N_4_ would be occupied by dissociated H_2_O in solution. Therefore, the PM-C_3_N_4_ with OH absorbing at the PM_1_ site was taken as the initial state of OER, and the adsorption energy for the *OH is shown in Figure 2b. For other intermediates, various adsorption sites were compared to determine the most stable configuration, and the most stable configuration is shown in Figures S2–S9.

**Figure 3:**
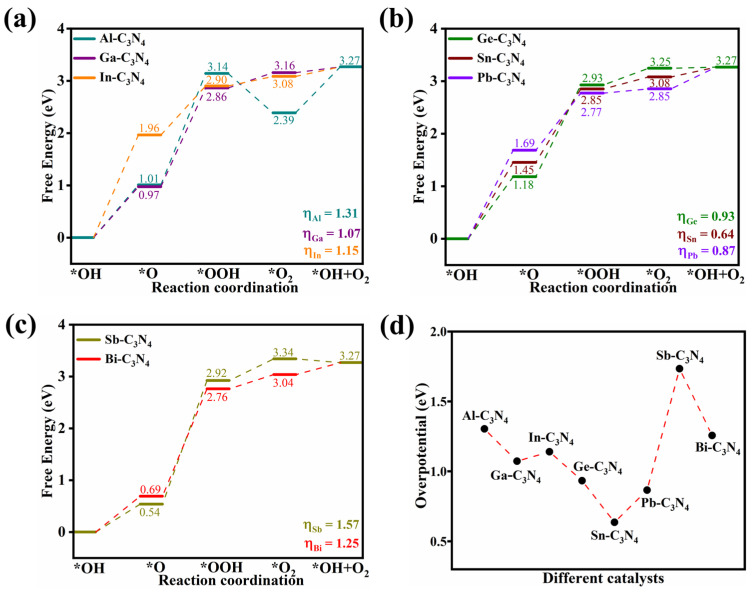
Reaction free energy profiles of the OER on PM–C_3_N_4_, (**a**) PM = Al, Ga, In; (**b**) PM = Ge, Sn, Pb; and (**c**) PM = Sb, Bi at *U* = 0 V and pH = 7. (**d**) The overpotential of OER catalyzed by PM-C_3_N_4_.

Figure 3a–c depicts the free energy diagram of OER catalyzed by the III A (Al, Ga and In), IV A (Ge, Sn and Pb) and V A (Sb and Bi) groups PM supported by *g*-C_3_N_4_, respectively. Results show that all the steps are uphill in the absence of photovoltage. For PM = Al and Ga in III A group (Figure 3a), the formation of *OOH intermediate encounters the largest ∆*G*, resulting in the overpotential of 1.31 and 1.07 eV, respectively. For In-C_3_N_4_, the overpotential is 1.15 eV, which was contributed by the process of *OH to *O. For the PM in IV A group (Ge, Sn and Pb), the largest ∆*G* among the whole process for Ge-C_3_N_4_ is the transformation from *O intermediate to *OOH intermediate with an overpotential of 0.93 eV, whereas the transformation from *OH intermediate to *O intermediate for Sn-C_3_N_4_ and Pb-C_3_N_4_, with overpotentials of 0.64 and 0.87 eV, respectively. For the PM in V A group (Sb and Bi), for both, the largest ∆*G* of them is the second step (*O to *OOH) with overpotentials of 1.57 and 1.25 eV, respectively.

Overall, the overpotentials of all PM-C_3_N_4_ are shown in Figure 3d, and it can be observed that the overpotentials of OER catalyzed by PM-C_3_N_4_ are relatively low while PM = Ge, Sn and Pb, thus being good candidates that possess potential photocatalytic reactivity towards OER.

#### 2.2.2. The HER Reactivity

Consequently, we investigated another half-reaction HER for photocatalytic water splitting on PM-C_3_N_4_. The HER in overall water splitting can be expressed as follows:

4H^+^ + 4e^−^ → 2H_2_, (7)

The above equation can be further divided into the following two one-electron primitive steps:

* + H^+^ + e^−^ → *H, (8)

*H + H^+^ + e^−^ → * + H_2_, (9)

**Figure 4:**
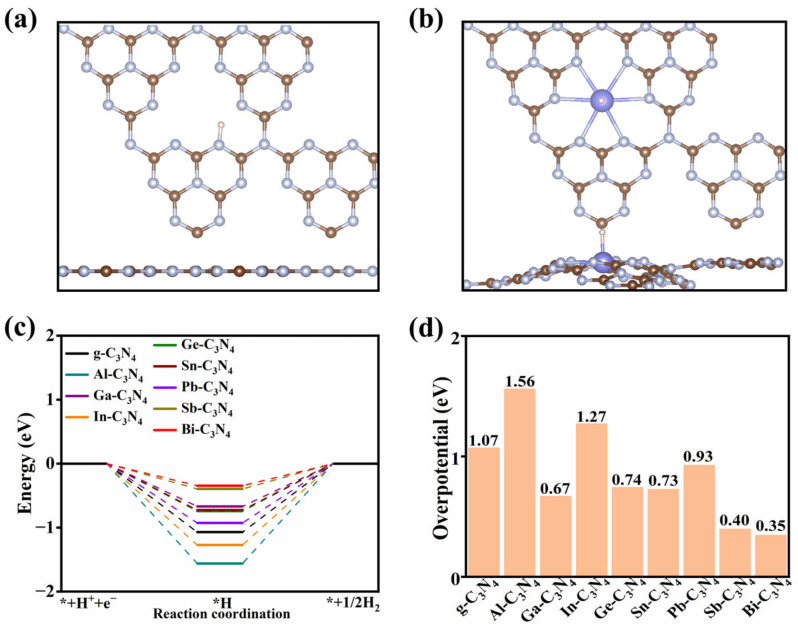
(**a**) Top view and side views of the H adsorption structure on *g*-C_3_N_4_. (**b**) Top view and side views of the H adsorption structure on PM-C_3_N_4_. Color scheme: greyish-white, nitrogen; brown, carbon; white, hydrogen; blue, PM_1_. (**c**) Free energy diagram for the reaction of *g*-C_3_N_4_ and PM-C_3_N_4_ at *U* = 0 V vs. RHE. (**d**) Overpotentials of HER reaction on *g*-C_3_N_4_ and PM-C_3_N_4_.

Given the known HER activity of pristine *g*-C_3_N_4_, it was selected as a reference for this investigation. When the H intermediate adsorbs onto *g*-C_3_N_4_, it tends to anchor at the nitrogen site of the intrinsic vacancies of *g*-C_3_N_4_, as depicted in Figure 4a. In contrast, for the PM-C_3_N_4_, they preferentially attach to the PM, causing the C_3_N_4_ framework undergoing a transformation from a planar to a corrugated structure, as depicted in Figure 4b. Figure S10 displays the optimized configurations of *H on these PM-C_3_N_4_ substrates. The Gibbs free energy changes and overpotentials during the HER process for various PM-C_3_N_4_ are illustrated in Figure 4c,d. These results indicate that the undoped *g*-C_3_N_4_ has an overpotential of 1.07 eV during the HER process, which increases upon doping with Al and In, thereby inhibiting HER. Conversely, the incorporation of PMs such as Ga, Ge, Sn, Pb, Sb and Bi reduces the overpotential and enhances the HER reactivity.

### 2.3. Electronic Structure of Candidates

**Figure 5:**
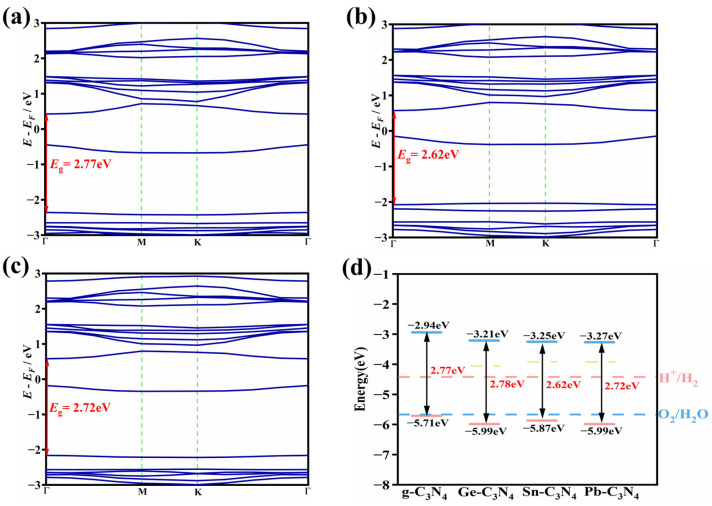
The electronic band structures of (**a**) Ge-C_3_N_4_, (**b**) Sn-C_3_N_4_ and (**c**) Pb-C_3_N_4_; (**d**) positions of band edge for *g*-C_3_N_4_, Ge-C_3_N_4_, Sn-C_3_N_4_ and Pb-C_3_N_4_ against the redox potentials of water splitting. The green dashed lines represent the impurity energy level.

As mentioned above, PM-C_3_N_4_ (PM = Ge, Sn and Pb) performs better in HER and OER in the absence of photo-irradiation. To assess the photocatalytic reactivity of these candidates towards both OER and HER and to elucidate the modulation effect of PM_1_ on the electronic structure of the pure *g*-C_3_N_4_, we computed their electronic band structures utilizing the HSE06 functional (Figure 5a–c and Figure S11). The findings reveal that the undoped *g*-C_3_N_4_ is a semiconductor with an indirect *E*_g_ of 2.77 eV (Figure S11a), which is in good agreement with the computational and experimental values previously reported [33,48]. Electronic band structures of Ge-, Sn- and Pb-C_3_N_4_ are shown in Figure 5a–c, respectively. All of them have a direct *E*_g_, with the *E*_g_ of 2.78, 2.62 and 2.72 eV, respectively. Furthermore, doping *g*-C_3_N_4_ with PM_1_ introduces impurity energy levels near the Fermi level. For Ge-C_3_N_4_, Sn-C_3_N_4_ and Pb-C_3_N_4_, their impurity energy levels are closer to the CBM and higher than the reduction potentials for photocatalytic water splitting. This allows the impurity energy levels to serve as stepping stones for photogenerated electrons/holes, thereby enhancing the photocatalytic ability of the catalysts.

The relative position of CBM and VBM in relation to the redox potentials of two half-reactions dictates the driving force of photoinduced electron/hole. In other words, the CBM of the catalysts should be higher than the redox potential of HER, while the VBM should be lower than the redox potential of OER. As shown in Figure 5d, all three catalysts, Ge-C_3_N_4_, Sn-C_3_N_4_ and Pb-C_3_N_4_, exhibit a downshift in both VBM and CBM. However, they remain above the redox potential for overall water splitting. This suggests that some of the initially strong driving force for HER is slightly compromised to enhance the driving force for OER after the introduction of Ge_1_, Sn_1_ or Pb_1_ into *g*-C_3_N_4_.

**Figure 6:**
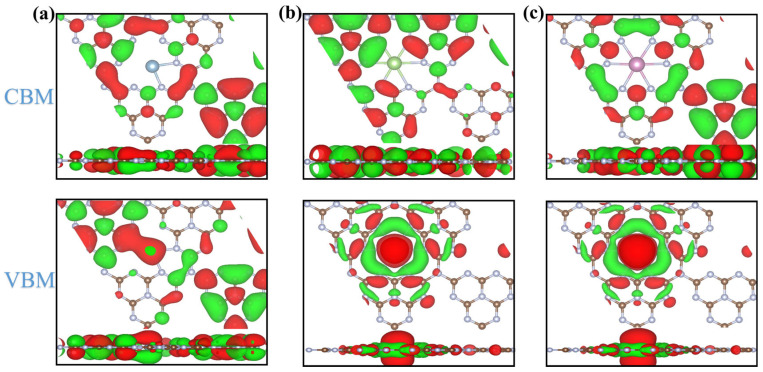
The band-decomposed electron density isosurface of the CBM (upper) and the VBM (bottom) of PM-C_3_N_4_ (PM = Ge, Sn and Pb) with the isolevel of 0.008 e/Å^3^. (**a**) CBM and VBM for the top view and side view of Ge-C_3_N_4_, respectively; (**b**) CBM and VBM for the top view and side view of Sn-C_3_N_4_, respectively; (**c**) CBM and VBM for the top view and side view of Pb-C_3_N_4_, respectively.

Subsequently, the electron density distribution for the CBM and VBM of PM-C_3_N_4_ (PM = Ge, Sn and Pb) was examined, as depicted in Figure 6. The VBM of PM-C_3_N_4_ is localized at the PM and its adjacent regions, whereas the CBM is primarily attributed to the *g*-C_3_N_4_ framework alone, which is consistent with previous studies [49]. This trend is observed across PM-C_3_N_4_ materials with PM = Al, Ga, In, Sb and Bi, as shown in Figures S13 and S14. The spatial separation of photogenerated electrons and holes is facilitated by the distribution of VBM and CBM in PM-C_3_N_4_, thereby inhibiting their recombination. The incorporation of PM not only alters the electronic structure of PM-C_3_N_4_ but also encourages charge separation between carriers. This finding is significant for the design and preparation of highly efficient photocatalysts.

## 3. Conclusions

In this study, we systematically investigate the regulatory effect of PM to *g*-C_3_N_4_ on the photocatalytic performance of water splitting by DFT calculations. By analyzing the calculated energy diagrams for both HER and OER, we identify PM-C_3_N_4_ (PM = Ge, Sn and Pb) as promising candidates for overall photocatalytic water splitting. When PM (specifically Ga, Ge and Sn) is introduced, the band gaps of PM-C_3_N_4_ undergo slight changes. This adjustment ensures ample driving force for OER while sacrificing a small portion of the originally strong HER driving force. Consequently, the photocatalytic activity for overall water splitting is enhanced. Notably, the electron density plots of the VBM and CBM for PM-C_3_N_4_ reveal their localization in different regions, spatially separating them and inhibiting carrier recombination. Overall, modifying *g*-C_3_N_4_ with PM (Ge, Sn and Pb) not only reduces the corresponding overpotentials for OER and HER but also tunes the electronic structure and carrier driving forces, leading to highly efficient photocatalysts. These insights have significant implications for the design and synthesis of advanced materials in this field.

## 4. Computational Method

All spin-polarized calculations were performed using the Vienna ab initio simulation package (VASP) based on plane–wave basis sets [50]. Electron–ion interactions were modeled using projector augmented wave (PAW) potentials with a plane–wave energy cutoff of 400 eV [51,52]. The generalized gradient approximation (GGA) with the Perdew−Burke−Ernzerhof (PBE) functional was adopted for describing the interaction between electrons [53]. The Heyd−Scuseria−Ernzerhof screened hybrid density functional method (HSE06) was employed to obtain the electronic structures owing to its well-known underestimation by pure GGA in terms of band gap [54]. The Brillouin zone was sampled using the 3 × 3 × 1 and 9 × 9 × 1 *k*-points grids of the Monkhorst–Pack scheme for structure relaxations and electronic structure computations, respectively [55]. Van der Waals interactions were described by the DFT-D3 method throughout all calculations. The convergence criteria for energy and force were set to 10^−5^ eV and 0.03 eV/Å, respectively. A 2 × 2 × 1 supercell of *g*-C_3_N_4_ was modeled to support dopants, while a vacuum layer of 20 Å along the *z*-axis was adopted to avoid the interaction between periodic boundaries [25].

The Gibbs free energy diagrams of two half-reactions of photocatalytic water splitting were obtained via Nørskov’s computational hydrogen electrode (CHE) model [56], in which the changes of free energy (Δ*G*) for each elementary reaction step were calculated as follows:

$$∆G=∆E+∆E_{ZPE}-T∆S-eU+∆G_{pH}$$

where the ∆E, $∆E_{ZPE}$ and ∆S is the change of electronic energy, zero-point energy and entropy of elementary reaction steps, respectively; *T* is set to 298.15 K; e is the elementary electron charge; U is electrode potential with respect to the standard hydrogen electrode (SHE), which is 0 V without photo-irradiation; $∆G_{pH}$ is the free energy differences contributed by protons ($∆G_{pH}=0.059\times pH$). The free energy (H^+^ + e^−^) of proton-electron pair is taken as 1/2$G_{H_{2}}$ under standard conditions while the free energy of O_2_ was derived as $\;G_{O_{2}}=2G_{H_{2}O}-2G_{H_{2}}-4.92$ [47]. The overpotential of the OER (*η_OER_*) can be used as an indicator to evaluate the activity of the catalyst, with a lower *η_OER_* value indicating stronger OER activity. The definition of theoretical *η_OER_* is:

$$\eta_{OER}=\frac{\max\left({∆G}_{pH=0}\right)}{e}-1.23$$

which is independent of the pH [57]. In addition, to evaluate the stabilities of anchored PM_1_, the difference between $E_{b}$ and $E_{c}$ was analyzed [58,59]. The binding energies ($E_{b}$) of different metal single atoms anchoring on *g*-C_3_N_4_ and corresponding cohesive energy ($E_{c}$) are defined as:

$$E_{b}=E_{PM-C_{3}N_{4}}-E_{g-C_{3}N_{4}}-E_{PM}$$

$$E_{c}=\frac{E_{PM-bulk}}{n}-E_{PM}$$

where $E_{PM-C_{3}N_{4}}$, $E_{g-C_{3}N_{4}}$ and $E_{PM}\;$ were the calculated energy of PM-C_3_N_4_, *g*-C_3_N_4_ substrate and isolated PM_1_, respectively.

## Data Availability

Data are contained within the article and Supplementary Materials.

## Supplementary Materials

The following supporting informatiodn can be downloaded at: https://www.mdpi.com/article/10.3390/molecules29092030/s1, Figure S1: Optimized structures of PM-C_3_N_4_ (PM = Al, Ga, In, Tl, Ge, Sn, Pb, Sb, Bi); Figure S2: Optimized structures for intermediates during OER catalyzed by Al-C_3_N_4_; Figure S3: Optimized structures for intermediates during OER catalyzed by Ga-C_3_N_4_; Figure S4: Optimized structures for intermediates during OER catalyzed by In-C_3_N_4_; Figure S5: Optimized structures for intermediates during OER catalyzed by Ge-C_3_N_4_; Figure S6: Optimized structures for intermediates during OER catalyzed by Sn-C_3_N_4_; Figure S7: Optimized structures for intermediates during OER catalyzed by Pb-C_3_N_4_; Figure S8: Optimized structures for intermediates during OER catalyzed by Sb-C_3_N_4_; Figure S9: Optimized structures for intermediates during OER catalyzed by Bi-C_3_N_4_; Figure S10: Optimized structures for *H on *g*-C_3_N_4_ and PM-C_3_N_4_; Figure S11: The electronic band structures of *g*-C_3_N_4_ and PM-C_3_N_4_; Figure S12: Band edges (i.e., VBM and CBM) alignment of *g*-C_3_N_4_ and PM-C_3_N_4_ corresponding to the redox potential for water splitting; Figure S13: The isosurface (isolevel: 0.008 e/Å^3^) of band-decomposed electron density for the CBM (upper) and VBM (bottom) of PM-C_3_N_4_ (PM = Al, Ga, In); Figure S14: The isosurface (isolevel: 0.008 e/Å^3^) of band-decomposed electron density for the CBM (upper) and VBM (bottom) of PM-C_3_N_4_ (PM = Sn, Bi); Table S1: The average bond length between the PM and nitrogen atoms in PM-C_3_N_4_.
