# Supplementary material for: Theoretical Study of p-Block Metal Single-Atom-Loaded Carbon Nitride Catalyst for Photocatalytic Water Splitting
Source: Molecules. 2024 Apr 28;29(9):2030. doi: 10.3390/molecules29092030 (PMC11085354; doi:10.3390/molecules29092030)
Supplement: Supplementary file 1 [file molecules-29-02030-s001.zip › molecules-2982777-supplementary.pdf]

Supplementary information

Theoretical study of *p*-block metal single atom loaded carbon nitride catalyst for photocatalytic water splitting

Mengning Chen [1], Yidi Wu [1], Qiang Wan [2,*] and Sen Lin [1,*]

1 State Key Laboratory of Photocatalysis on Energy and Environment, Fuzhou University, Fuzhou, 350116, China

2 Key Laboratory of the Ministry of Education for Advanced Catalysis Materials, Institute of Physical Chemistry, Zhejiang Normal University, Jinhua, 321004, China

E-mail: slin@fzu.edu.cn; qwan@zjnu.edu.cn

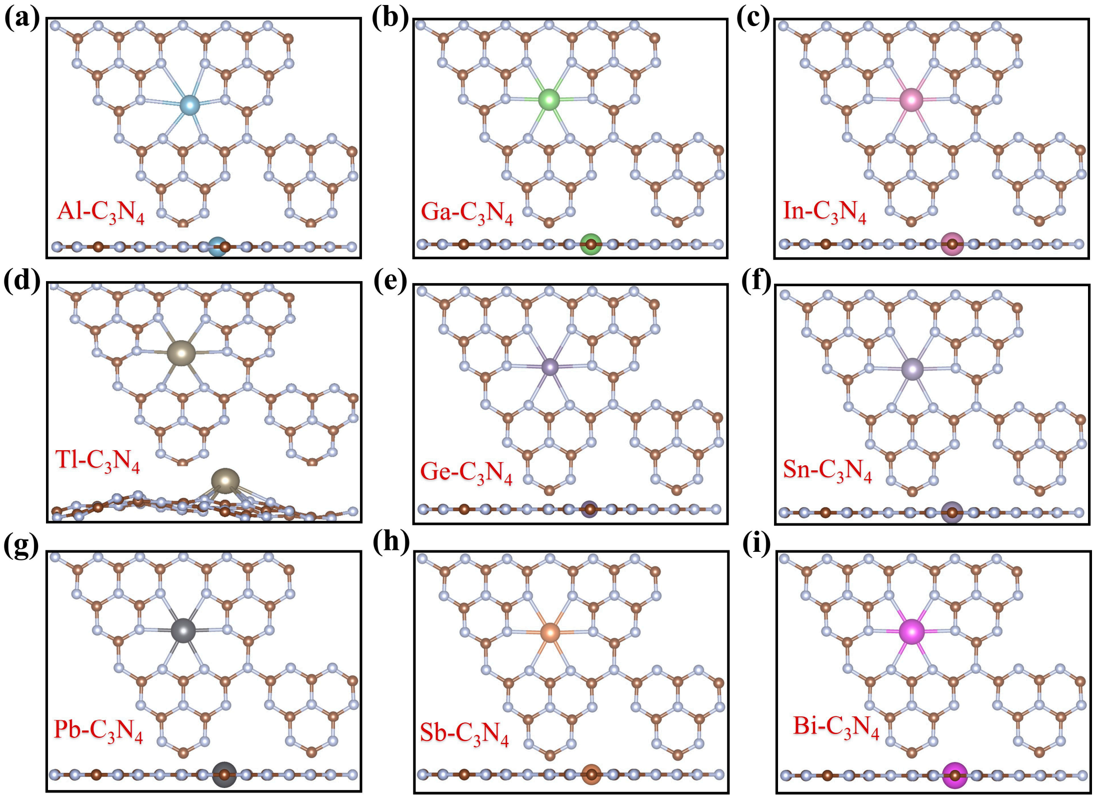

Figure S1. Optimized structures of PM-C_3N_4 (PM = Al, Ga, In, Tl, Ge, Sn, Pb, Sb, Bi). Color scheme: brown, carbon; greyish-white, nitrogen.

Table S1. The average bond length between the PM and nitrogen atoms in PM-C_3N_4.

	p-block metal (PM)	L(PM-N) (Å)
The IIIA group	Al	2.39
	Ga	2.40
	In	2.42
The IVA group	Ge	2.35
	Sn	2.42
	Pb	2.44
The VA group	Sb	2.40
	Bi	2.43

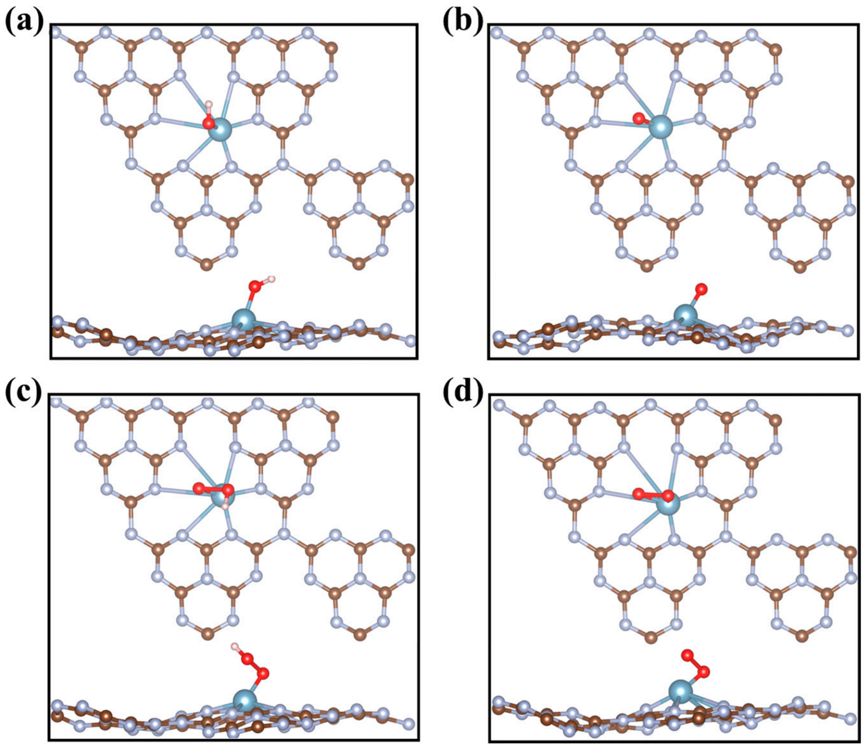

Figure S2. Optimized structures for intermediates during OER catalyzed by Al-C_3N_4. (a) *OH; (b) *O; (c) *OOH; (d) *OO intermediates. Color scheme: red, oxygen.

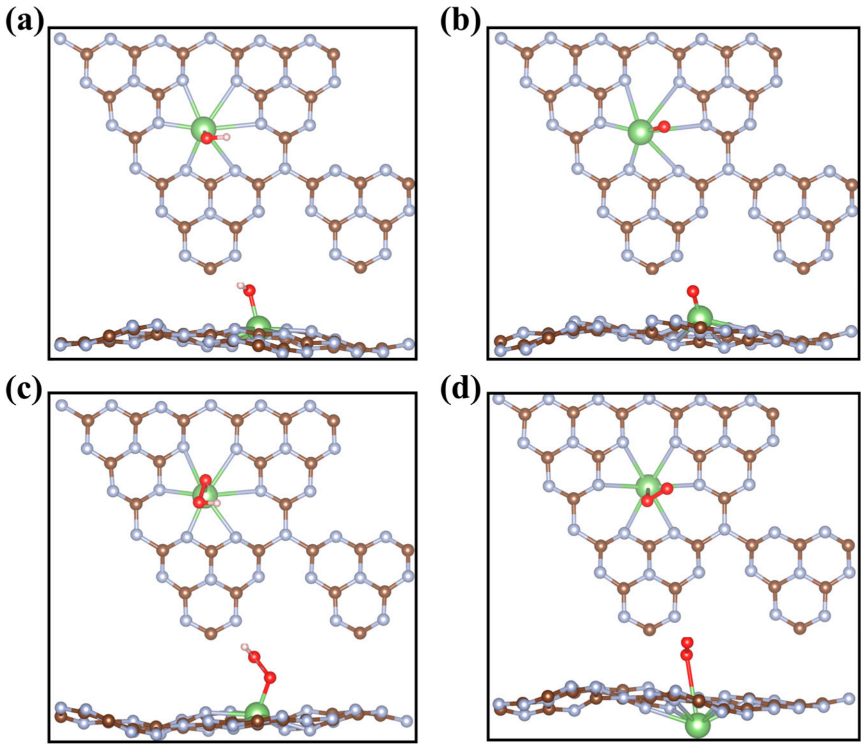

Figure S3. Optimized structures for intermediates during OER catalyzed by Ga-C_3N_4. (a) *OH; (b) *O; (c) *OOH; (d) *OO intermediates.

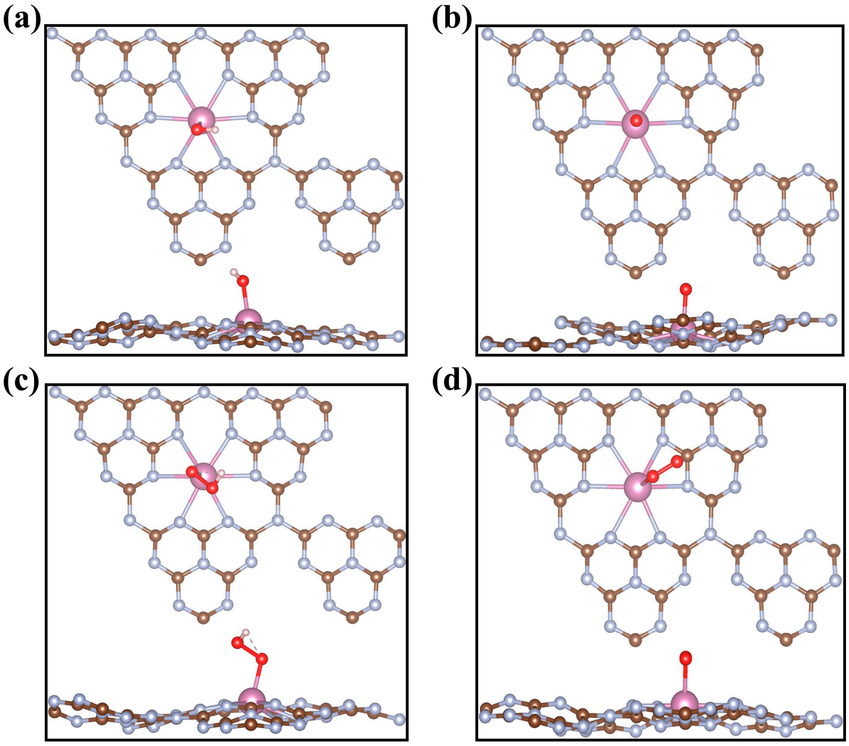

Figure S4. Optimized structures for intermediates during OER catalyzed by In-C_3N_4. (a) *OH; (b) *O; (c) *OOH; (d) *OO intermediates.

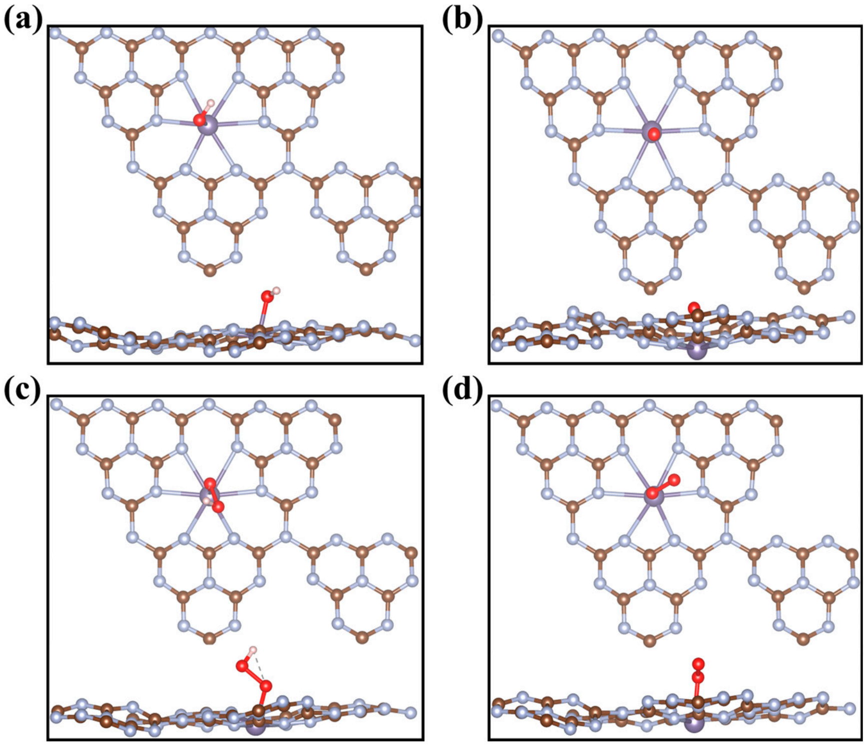

Figure S5. Optimized structures for intermediates during OER catalyzed by Ge-C_3N_4. (a) *OH; (b) *O; (c) *OOH; (d) *OO intermediates.

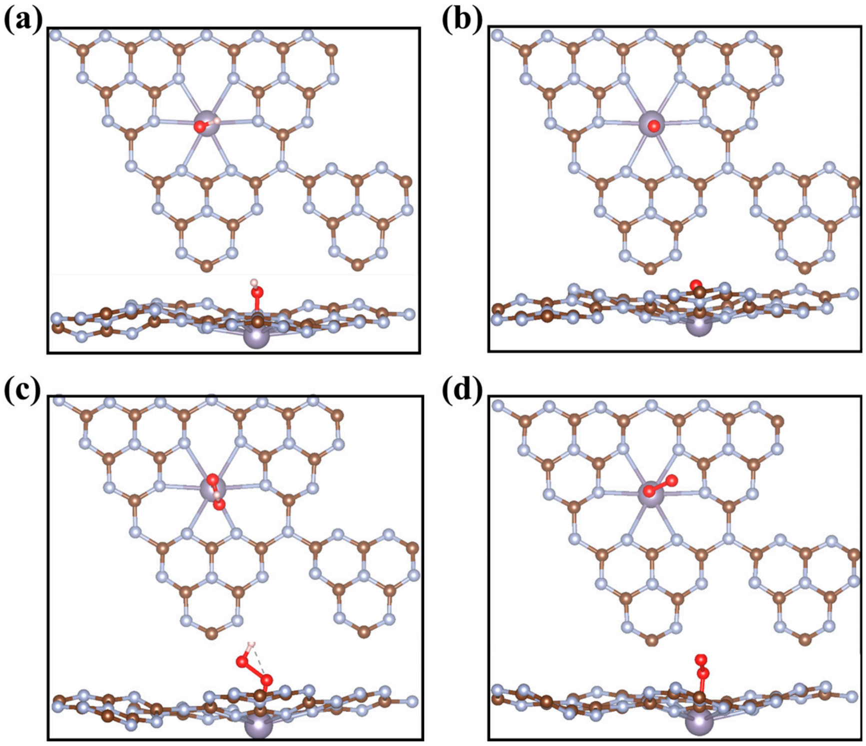

Figure S6. Optimized structures for intermediates during OER catalyzed by Sn-C_3N_4. (a) *OH; (b) *O; (c) *OOH; (d) *OO intermediates.

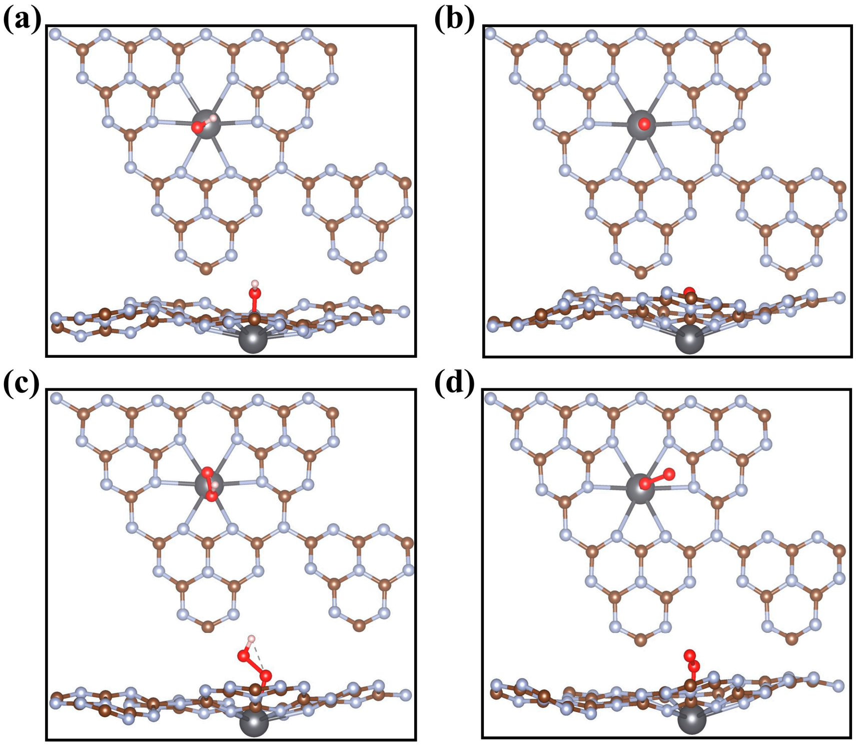

Figure S7. Optimized structures for intermediates during OER catalyzed by Pb-C_3N_4. (a) *OH; (b) *O; (c) *OOH; (d) *OO intermediates.

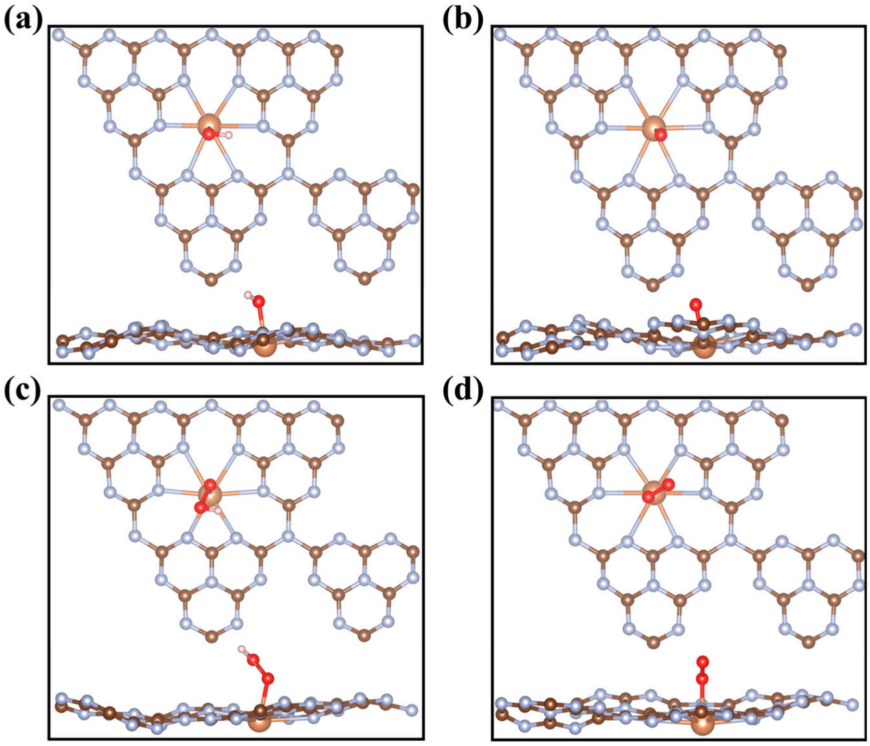

Figure S8. Optimized structures for intermediates during OER catalyzed by Sb-C_3N_4. (a) *OH; (b) *O; (c) *OOH; (d) *OO intermediates.

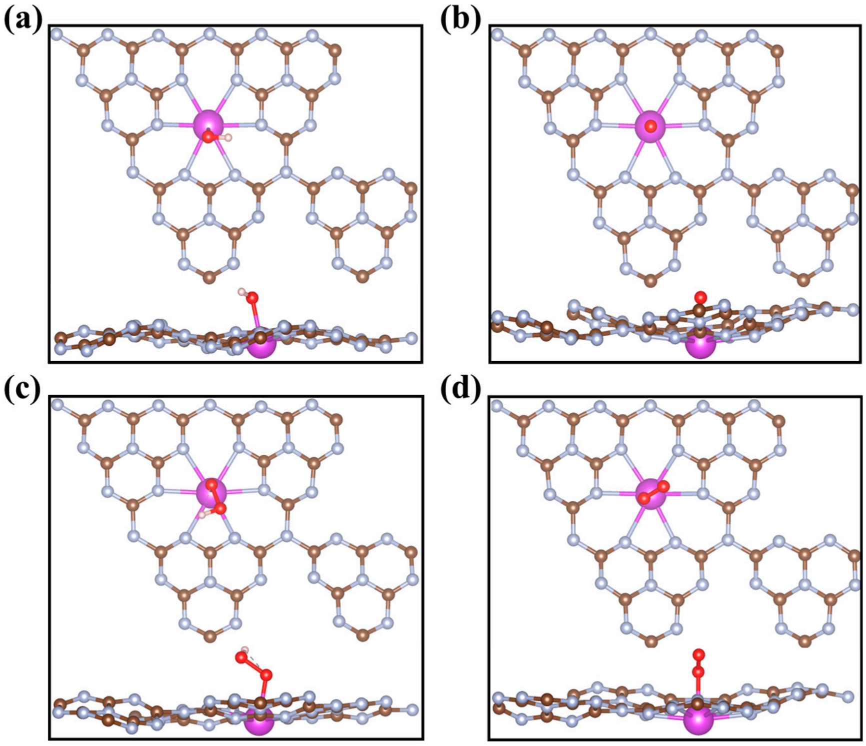

Figure S9. Optimized structures for intermediates during OER catalyzed by Bi-C_3N_4. (a) *OH; (b) *O; (c) *OOH; (d) *OO intermediates.

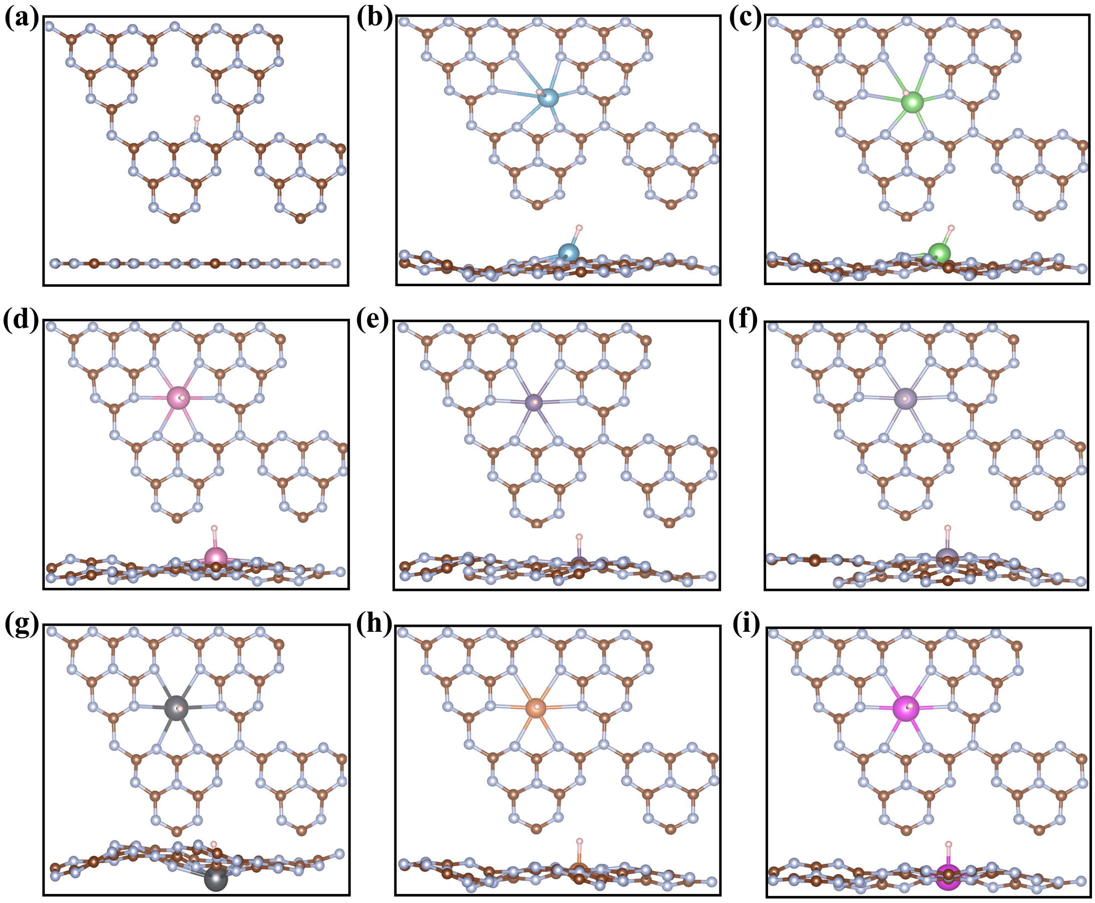

Figure S10. Optimized structures for *H on *g*-C_3N_4 and PM-C_3N_4. (a) *g*-C_3N_4; (b) Al-C_3N_4; (c) Ga-C_3N_4; (d) In-C_3N_4; (e) Ge-C_3N_4; (f) Sn-C_3N_4; (g) Pb-C_3N_4; (h) Sb-C_3N_4 and (i) Bi-C_3N_4.

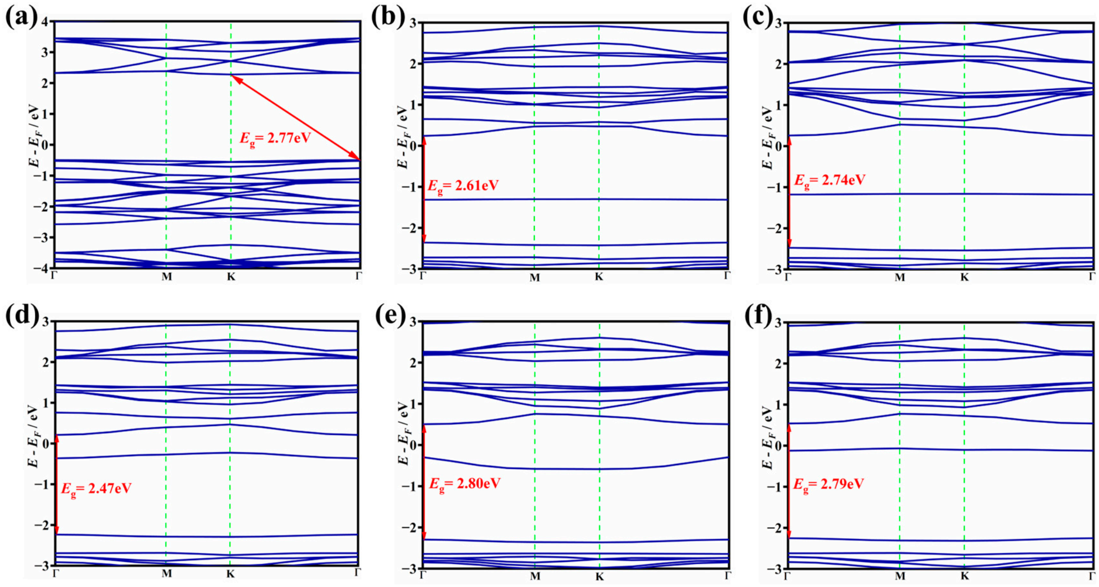

Figure S11. The electronic band structures of *g*-C_3N_4 and PM-C_3N_4. (a) *g*-C_3N_4; (b) Al-C_3N_4; (c) Ga-C_3N_4; (d) In-C_3N_4; (e) Sb-C_3N_4 and (f) Bi-C_3N_4.

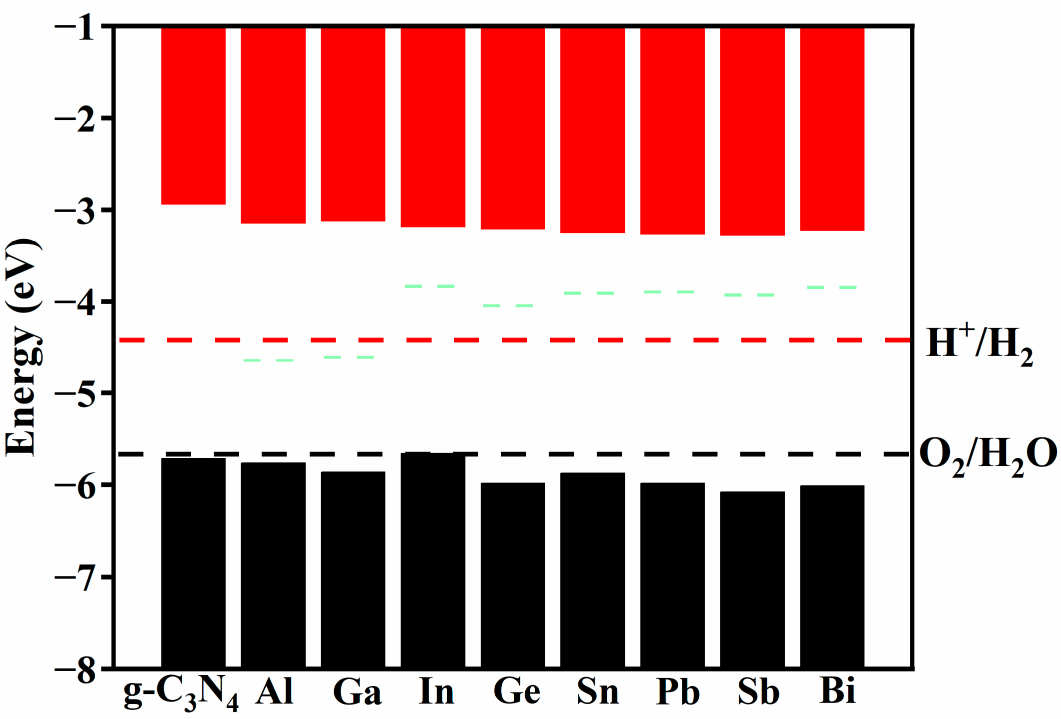

Figure S12. Band edges (i.e., VBM and CBM) alignment of *g*-C_3N_4 and PM-C_3N_4 corresponding to the redox potential for water splitting. The green dash-lines represent the doping energy level.

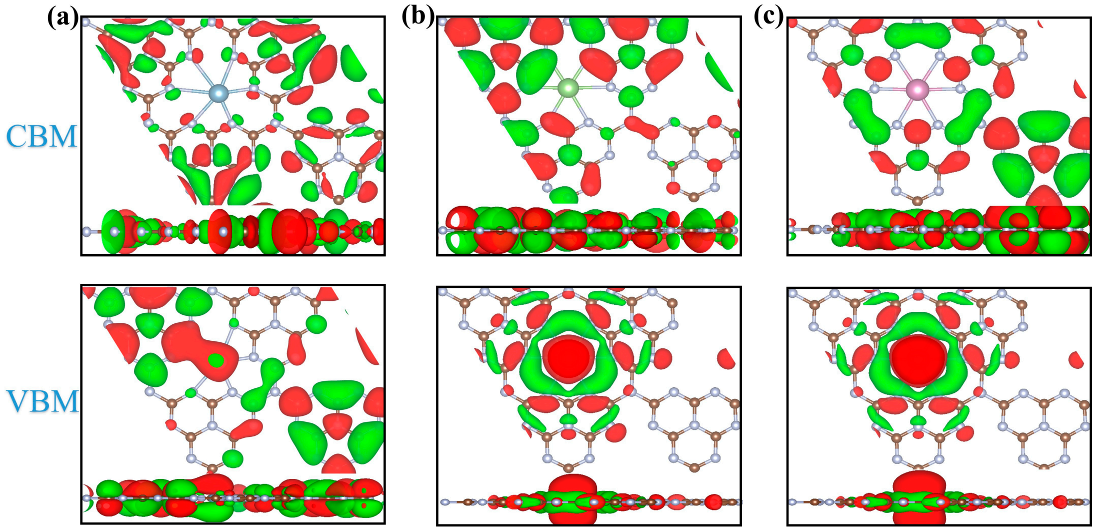

Figure S13. The isosurface (isolevel: 0.008 e/Å^3) of band-decomposed electron density for the CBM (upper) and VBM (bottom) of PM-C_3N_4 (PM=Al, Ga, In). (a) Al-C_3N_4; (b) Ga-C_3N_4 and (c) In-C_3N_4.

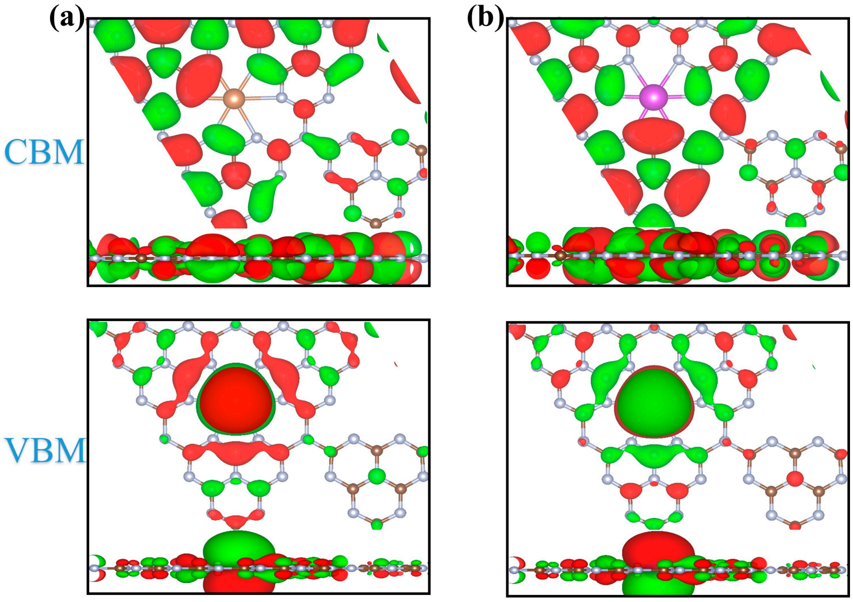

Figure S14. The isosurface (isolevel: 0.008 e/Å^3) of band-decomposed electron density for the CBM (upper) and VBM (bottom) of PM-C_3N_4 (PM=Sn, Bi). (a) Sn-C_3N_4 and (b) Bi-C_3N_4.
